# International Scientific Collaboration in HIV and HPV: A Network Analysis

**DOI:** 10.1371/journal.pone.0093376

**Published:** 2014-03-28

**Authors:** Tazio Vanni, Marco Mesa-Frias, Ruben Sanchez-Garcia, Rafael Roesler, Gilberto Schwartsmann, Marcelo Z. Goldani, Anna M. Foss

**Affiliations:** 1 National Institute of Science and Technology in Translational Medicine, Hospital de Clínicas de Porto Alegre, Porto Alegre, Brazil; 2 Centre for the Mathematical Modelling of Infectious Diseases, London School of Hygiene and Tropical Medicine, London, United Kingdom; 3 Mathematical Sciences, University of Southhampton, Southhampton, United Kingdom; 4 Laboratory of Neuropharmacology and Neural Tumor Biology, Department of Pharmacology, Institute for Basic Health Sciences, Federal University of Rio Grande do Sul, Porto Alegre, Brazil; 5 Cancer Research Laboratory, University Hospital Research Center (CPE-HCPA), Federal University of Rio Grande do Sul, Porto Alegre, Brazil; 6 Faculdade de Medicina e Hospital de Clínicas de Porto Alegre, Universidade Federal do Rio Grande do Sul, Porto Alegre, Brazil; 7 Social and Mathematical Epidemiology Research Group, Faculty of Public Health and Policy, London School of Hygiene and Tropical Medicine, London, United Kingdom; 8 Visitor to the Academic Unit of Primary Care and Population Sciences, Faculty of Medicine, University of Southampton, Southampton, United Kingdom; Baylor College of Medicine, United States of America

## Abstract

Research endeavours require the collaborative effort of an increasing number of individuals. International scientific collaborations are particularly important for HIV and HPV co-infection studies, since the burden of disease is rising in developing countries, but most experts and research funds are found in developed countries, where the prevalence of HIV is low. The objective of our study was to investigate patterns of international scientific collaboration in HIV and HPV research using social network analysis. Through a systematic review of the literature, we obtained epidemiological data, as well as data on countries and authors involved in co-infection studies. The collaboration network was analysed in respect to the following: centrality, density, modularity, connected components, distance, clustering and spectral clustering. We observed that for many low- and middle-income countries there were no epidemiological estimates of HPV infection of the cervix among HIV-infected individuals. Most studies found only involved researchers from the same country (64%). Studies derived from international collaborations including high-income countries and either low- or middle-income countries had on average three times larger sample sizes than those including only high-income countries or low-income countries. The high global clustering coefficient (0.9) coupled with a short average distance between researchers (4.34) suggests a “small-world phenomenon.” Researchers from high-income countries seem to have higher degree centrality and tend to cluster together in densely connected communities. We found a large well-connected community, which encompasses 70% of researchers, and 49 other small isolated communities. Our findings suggest that in the field of HIV and HPV, there seems to be both room and incentives for researchers to engage in collaborations between countries of different income-level. Through international collaboration resources available to researchers in high-income countries can be efficiently used to enroll more participants in low- and middle-income countries.

## Introduction

As science evolves, important scientific achievements require the collaborative effort of an increasing number of researchers. The study of patterns of scientific collaboration allows us to gain further understanding of innovation and knowledge production. Scientific collaboration networks have been the subject of growing interest in the past few years [1]–[4]. Collaborative scientific publications have a long history. The first collaborative research paper was published in 1665 in the Philosophical Transactions of the Royal Society [5]. To date, the most multi-authored scientific paper was published in Physics Letters B in 2010, when 3,222 researchers from 32 different countries contributed to a study of ‘charged-particle multiplicities’ performed in the Large Hadron Collider at CERN [6].

No single researcher has all the means to conduct large epidemiological studies. Scientific collaboration is a critical tool for progress in epidemiology as it allows the pooling of data, expertise and resources, promoting synergies in the production of knowledge. International scientific collaborations are particularly important in HIV and HPV co-infection studies. Even though the burden of disease related to the co-infection is rising in developing countries [7], most researchers and research funds are found in developed countries where initiatives to scale-up HIV screening and the use of combined antiretrovirals have contributed to substantially limit the HIV pandemic [8].

Cervical cancer is caused by HPV and it is the most common cause of cancer-related deaths among women in developing countries [8], [9]. Despite mounting evidence on interventions to prevent cervical cancer, there is limited information on the prevalence and incidence of HPV infection and cervical abnormalities in HIV-positive women worldwide, and how the natural history of HPV to cervical cancer is modified by HIV infection and antiretroviral treatment [10]–[13]. Gaining better understanding of the epidemiology and biology of HIV and HPV co-infection would allow us to tailor more efficient screening and vaccination strategies to prevent cervical cancer [14]–[16].

Despite the importance of scientific collaborations to medical studies, there are limited studies analysing these collaborations [17]–[19]. In particular, no peer-reviewed publications investigating international scientific collaboration in HIV and HPV research could be found in Medline, Embase, or Global Health databases. Therefore, the objective of this study is to evaluate patterns of international scientific collaboration in HIV and HPV epidemiological research.

## Materials and Methods

### Search Strategy and Data Extraction

This analysis is part of a broad effort to summarize all prevalence and incidence estimates for HPV infection, as well as cytological and histological cervical abnormalities in HIV-positive women in order to populate mathematical models. Based on search strategies used in previous studies [20], [21], we systematically reviewed PubMed, OVID Medline, Embase, and Global Health database using the following combined keywords (HIV OR human immunodeficiency virus) AND (HPV OR human papilloma virus OR human papillomavirus). The query yielded 2,934 records, of which 1,793 remained after the removal of duplicates. The inclusion criteria were: peer-reviewed journal article, original research, epidemiological studies on the prevalence and/or incidence of HPV infection in the cervix of HIV-infected women (i.e. cross-sectional and cohort studies), published from 01/01/1996 to 30/09/2012 (i.e. the HAART era). Non peer-reviewed reports, review articles, news articles, editorials, and conference abstracts were excluded. There were no language restrictions. By screening titles and abstracts, two independent reviewers identified 278 eligible articles for which the full papers were retrieved.

From the papers retrieved, we extracted data for year of publication, title, journal, number of patients enrolled, country from which the patients were recruited, authors' names, institutional affiliation and location (country), as well as, epidemiological data. Each paper with more than one author was considered to be a scientific collaboration. Papers co-authored by authors affiliated to institutions from different countries were considered an international scientific collaboration [17], [22]. Countries were classified according to the World Bank's three economic groups: low-, middle-, and high-income [22].

### Social network analysis

A social network is a set of social entities, such as individuals, presenting some pattern of relationship between them [23]. These networks are usually represented by graphs, where nodes symbolize social entities and edges (or links) connect nodes that are related to each other. The underlying patterns of organization of such networks are the object of study of social network analysis (SNA) [24]. Studies of social networks reflect not only our inherent interest in these patterns, but also the importance of these networks in the spread of information. A famous example is the study conducted by Stanley Milgram [25], in which randomly selected subjects from Nebraska were asked to get a letter to a target subject in Boston through chains of friends and acquaintances. Milgram found that on average it took six steps for the letters to reach the target. This finding became part of the popular culture through John Guarés play, *Six Degrees of Separation* [26], and it is interpreted as evidence of the “small-world phenomenon” [27].

In our study, we used network analysis to evaluate collaborative networks between countries and authors in HIV and HPV research worldwide. For this purpose, we developed a programme in C++ to rearrange the data extracted in mixing matrices, which was further analysed in MATLAB and Gephi. MATLAB is a numerical computing environment suitable for the manipulation and analysis of matrices. Gephi is open-source network analysis software for visualization and exploration of networks and complex systems [28]. The Fruchterman-Reingold forced-directed algorithm was used to define the network layout. It is a flexible algorithm that optimizes the arrangement of the nodes in an undirected graph based on the strength (force) of their connection [29]. An undirected network is one in which edges have no orientation. We produced two entities' undirected networks: countries and authors.

The degree of centrality is one of various types of measure of centrality, or importance, of an entity in a network. It is perhaps the most intuitive since it is the number of links that a node has [24]. Besides degree centrality, we also computed betweenness and PageRank centrality for the authors' network. Betweenness centrality can be described as the number of shortest paths between different nodes in the network that pass through the node in question. It is a more informative measure than just the node's degree of connectivity, since it also captures the importance of the node as a bridge in the transmission of information through the network [23]. PageRank is a measure of the influence of a node in which scores are assigned to all nodes in the network based on, for example, their degree of centrality, and nodes connected to high-scoring nodes will have higher PageRank measure than those connected to low-scoring nodes. It is named after its inventor, Larry Page, co-founder of Google, and it is popularly used to rank the relative importance of hyperlinked documents [30].

The authors' network statistics included average degree of centrality, degree of centrality distribution, density, modularity, connected components, diameter, average distance between nodes, clustering coefficient, as well as number and size of clusters. Density measures how well connected the nodes are in the network relative to the theoretically possible connections [24]. Modularity measures the division of a network into modules, or communities. Networks with high modularity have dense connections between the nodes within the same module but sparse connections between nodes in different modules [31]. A connected component of an undirected network is a sub-network in which any two nodes can reach each other by paths composed by one or more edges [32]. The diameter is the longest distance between any two nodes in the network. Two connected nodes have a distance of one [33]. The clustering coefficient measures the degree to which nodes are embedded in their neighbourhoods [24]. A high clustering coefficient, along with low average distance between nodes, can indicate a “small-world phenomenon” [27], [34].

## Results

**Figure 1:**
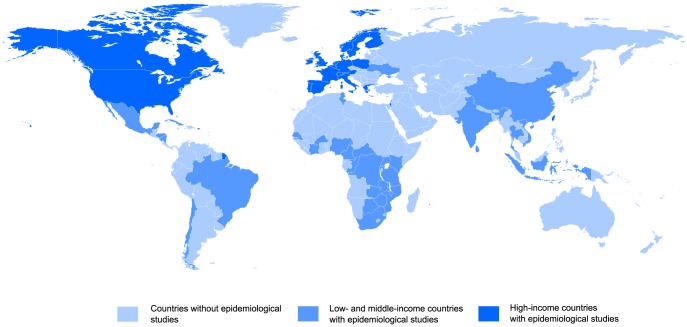
Countries from which participants were enrolled in HIV and HPV epidemiological studies.

Epidemiological studies on HIV and HPV could be found for most high-income countries (figure 1). However, there are still many low- and middle-income countries particularly in South America and Africa for which no study could be found. Table 1 indicates that most studies involved one country and eight or fewer researchers. The average number of entities per publication in the period studied (1996–2012) remained stable (data not shown). Table 2 shows that those studies involving international collaborations including high-income countries and either a low- or middle-income country had on average three times larger population sample sizes than those including only high-income countries or low-income countries.

**Table 1:** Size of collaborations* in respect to number of authors, institutions and countries

	Authors		Institutions		Countries	
Size of Collaboration	%	Absolute number	%	Absolute number	%	Absolute number
**1**	0.0	0	16.2	45	64.7	180
**2**	0.7	2	21.6	60	23.7	66
**3**	3.6	10	18.7	52	6.5	18
**4**	4.7	13	12.9	36	2.5	7
**5**	7.6	21	9.0	25	1.8	5
**6**	14.4	40	6.1	17	0.4	1
**7**	14.0	39	4.0	11	0.4	1
**8**	8.3	23	2.5	7	0.0	0
**9**	10.8	30	2.9	8	0.0	0
**10**	12.2	34	3.6	10	0.0	0
**11**	9.3	26	2.1	6	0.0	0
**12**	5.8	16	0.4	1	0.0	0
**13**	2.5	7	0.0	0	0.0	0
**14**	1.8	5	0.0	0	0.0	0
**≥15**	4.3	12	0.0	0	0.0	0

*Each paper with more than one author was considered to be a scientific collaboration.

**Table 2:** International collaboration by economic groups of countries (1996–2012)

Collaborating countries (1)	Collaborating countries (2)	Frequency	Average number of authors	Average number of institutions	Average number of countries	Average study sample size
High-income	Middle-income	49 (50%)	8.7	3.9	2.3	628
High-income	Low-income	36 (36.7%)	9.5	4.6	2.8	637
High-income	High-income	11 (11.3%)	9.6	5	2.5	186
Low-income	Low-income	2 (2%)	8.5	2.5	2.5	216

International collaborations were considered when the paper involved different countries, 98 in total. The combination of countries according to economic groups considered income extremes. For example, if there was a collaboration involving one high-income country and two middle-income countries, this was classified as a high- and middle-income country collaboration, not as middle and middle.

**Figure 2:**
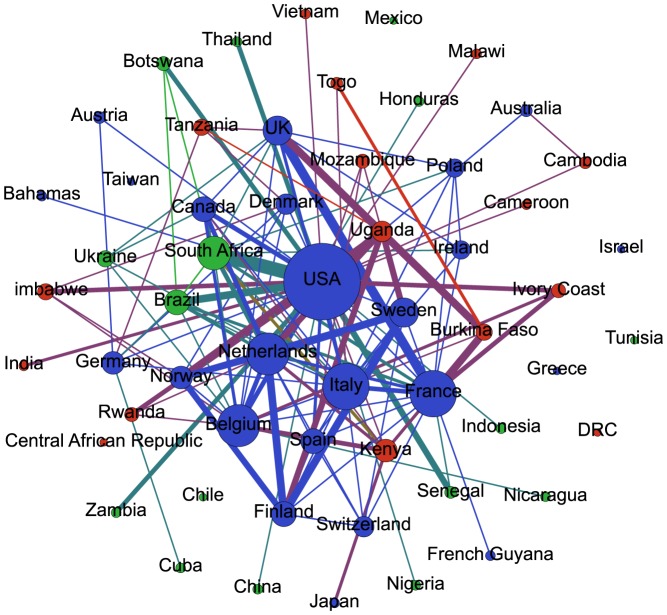
International network of scientific collaboration in HIV and HPV. High-income countries are in blue, middle-income countries in green and low-income countries in red. The colour of the edges was determined by the income-level of the countries linked, i.e. it is ‘sum’ of the colours of the nodes. Nodes were resized according to the degree of centrality. Edge width was defined according to the number of collaborations between the two countries.

The United States stands out as the country with the greatest number of international collaborations (Figure 2), particularly with South Africa, Uganda and Brazil. Despite the geographical proximity, collaboration between the US and Canada in HIV and HPV research was not very frequent. There was more intra-continental collaboration between European countries, including frequent collaboration between Norway, Sweden, Finland and the Netherlands, and with low-income countries like Uganda. The results indicate frequent collaborations between France and the United Kingdom and both countries collaborate with many other countries in Africa. Among middle-income countries, South Africa and Brazil stand out as the most collaborative countries. Among low-income countries, Kenya and Uganda were the most collaborative. We found many independent studies from low- and middle-income studies such as Democratic Republic of Congo, Central African Republic, Mexico and Chile.

**Figure 3:**
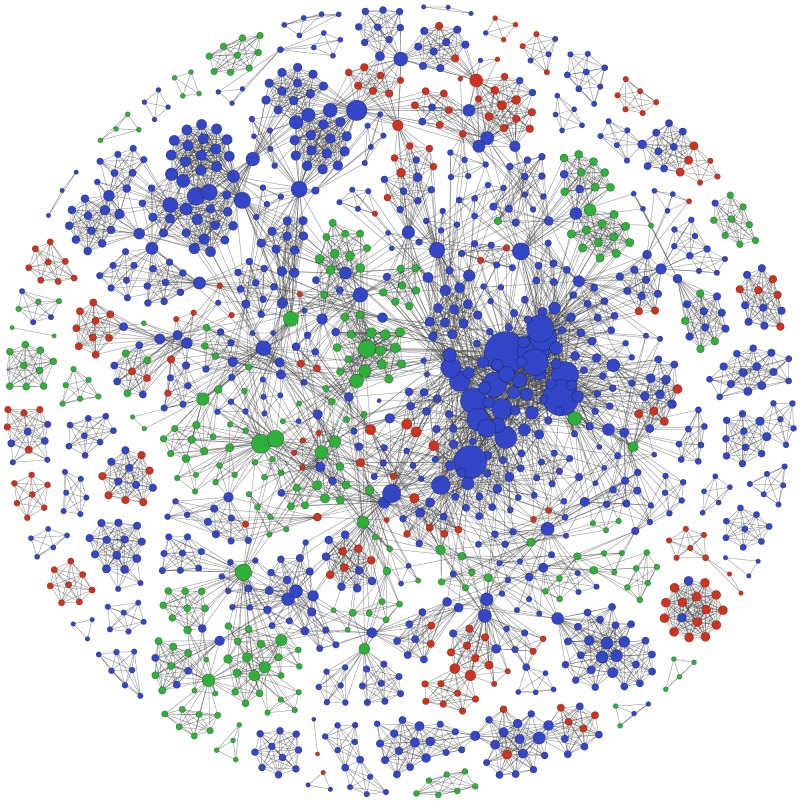
Co-authorship network according to the income-level of the country of origin (anonymized). Network composed of 1339 authors (or nodes). Authors from high-income countries are in blue, middle-income countries in green and low-income countries in red. Nodes were resized according to the degree of centrality.

In figure 3, we observe that most authors (or nodes) with the highest degree of centrality are from high-income countries. Some nodes from middle-income countries have a fairly high degree of centrality and among low-income countrieś nodes only one (near the top) has a higher degree. The average degree of centrality in the network was 11.1, meaning that on average authors had collaborated with 11 other authors in HIV and HPV research. The degree distribution followed a power law of exponent 2.5, which is different from a Poisson distribution found in randomly formed networks, and consistent with previous results for biomedical collaboration networks [2]. The high global clustering coefficient (0.9), associated with a short average distance between nodes (4.34), as well as diameter (9) suggest a “small-world phenomenon” within HIV and HPV researchers. The network has a low density of 0.008 reflecting its sparse connections. The largest connected component is composed of 949 nodes, which corresponds to 70% of the network. Besides the large connected component, there are 49 smaller components with sizes ranging from 2 to 42 nodes. Authors from countries within the same economic group often form these smaller components in the periphery of the network. However, collaborations between low- and high-income countries can also be found in the periphery and more rarely collaborations between middle- and high-income countries. We used Laplacian eigenvectors to identify clusters in the largest connected component [35], finding 11 clusters. They were: one core cluster of 276 nodes, two large clusters of 152 and 112 nodes and 8 smaller clusters. The core cluster can be seen almost in the centre of the largest connected component, including the nodes with the highest degree. The modularity was 0.85, which being a positive number supports the hypothesis that edges are not distributed at random.

**Figure 4:**
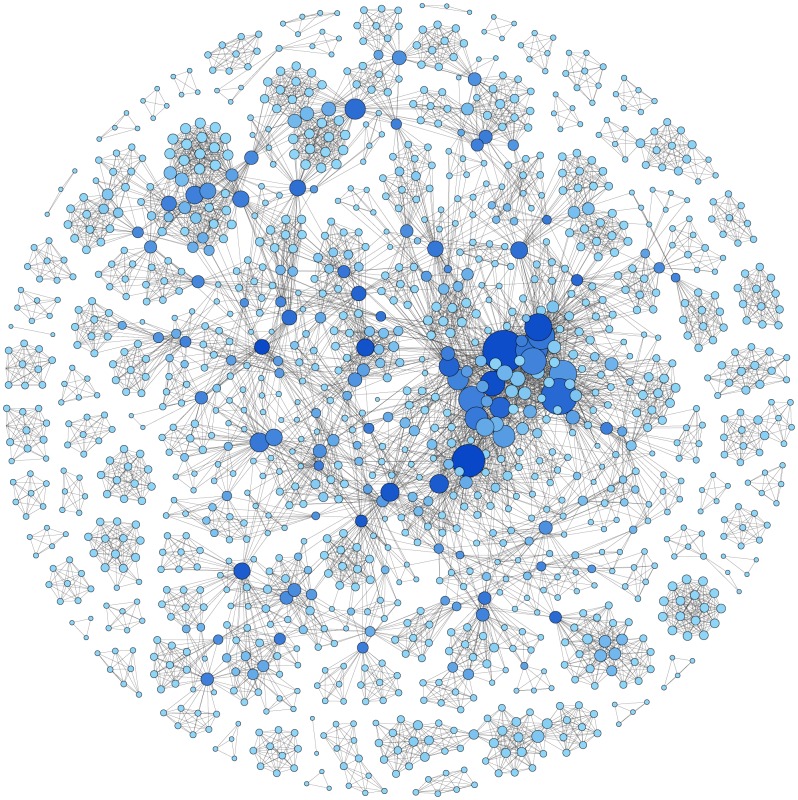
Co-authorship network according to betweenness centrality (anonymized). Network composed of 1339 authors (or nodes). Nodes were resized according to degree of centrality. The colour of the node was determined by its betweenness centrality. Dark blue nodes represent higher betweenness centrality. Conversely, light blue nodes represent lower betweenness centrality.

In Figure 4, outside the main cluster we can visualize some nodes in dark blue, which are smaller in size than other dark blue nodes in the main cluster. Although at a local level these nodes have limited importance (i.e. connectivity), at a global level they are important for bridging different groups of researchers. In Table 3, we can see the name of the most important authors in the network according to different metrics. According to degree of centrality, the 10 most important authors are all from high-income countries. When we consider betweenness centrality, some researchers from the International Agency for Research on Cancer (WHO) and middle-income countries also appear to have important positions in the collaboration network. Little difference can be found when comparing the degree of centrality and PageRank lists. It is worth noting that many of the best-ranked researchers in respect to degree of centrality and PageRank are affiliated to the Women's Interagency HIV Study.

**Table 3:** International scientific network (authors) statistics

Rank	Degree centrality	Authors (inc)	Betweenness centrality	Authors (inc)	PageRank	Authors (inc)
1.	113	Palefsky, J (3)	72,188	Shah, KV (3)	0.005	Palefsky, J (3)
2.	95	Burk, R (3)	59,863	Franceschi, S (3)	0.004	Burk, R (3)
3.	85	Shah, KV (3)	51,046	Palefsky, J (3)	0.004	Shah, KV (3)
4.	82	Minkoff, H (3)	49,355	Massad, LS (3)	0.003	Minkoff, H (3)
5.	69	Anastos, K (3)	48,743	Watts, DH (3)	0.003	Watts, DH (3)
6.	69	Watts, DH (3)	43,227	Levi, JE (2)	0.003	Cu-Uvin, S (3)
7.	68	Levine, AM (3)	37,387	Gravitt, PE (3)	0.003	Anastos, K (3)
8.	67	Cu-Uvin, S (3)	34,794	Gonçalves, M (2)	0.003	Strickler, HD (3)
9.	67	Strickler, HD (3)	33.644	Smith, JS (3)	0.003	Levine, AM (3)
10.	62	Massad, LS (3)	32,535	Lima, LP (2)	0.003	Williamson, S (2)

inc- income-level of the country of origin: (3) high-income, (2) middle-income and (1) low-income country. The definitions of the network statics here presented can be found in the Materials and Methods section, sub-section Social Network Analysis.

## Discussion

There are still many low- and middle-income countries for which no epidemiological estimates of HPV infection of the cervix among HIV-infected women could be found. The studies included in this analysis were highly collaborative in respect to the number of researchers involved but not as much in respect to the number of countries. Most studies only included researchers from the same country. Among studies involving international collaborations, those including high-income countries and either low- or middle-income countries seemed to have larger patient sample sizes than those including only high-income countries or low-income countries. This may be due to the leveraging of financial resources available to researchers in high-income countries and the larger patient populations in low- and middle-income countries, where the prevalence of HIV and HPV is higher.

The United States was the country with the largest number of international collaborations, particularly with South Africa, Uganda and Brazil. These three nations were the most collaborative among low- and middle-income countries. It is important to point out that densely linked networks are more resilient to the loss of central nodes. The high global clustering coefficient coupled with a short average distance between nodes suggests a “small-world phenomenon” among HIV and HPV researchers, similar to what was found by Newman et al in a general analysis of papers indexed in MEDLINE [2]. We found that the researchers from high-income countries seem to have a high number of research collaborations among them and to cluster together in densely connected communities, particularly those from the US. There is a large well-connected community, which encompasses 70% of researchers, and other much smaller communities. Some researchers from international institutions and middle-income countries play an important role by bridging different research communities in the network. The fact that many of the best-ranked researchers in respect to degree of centrality and PageRank are affiliated to the Women's Interagency HIV Study suggests that funding stream plays an important role in the network formation.

Although we did not find other studies on HIV and HPV research networks, we found a few scientometrics studies on HIV. These studies examining patterns in HIV research provided a base of understanding how a similar research field evolved. A citation analysis in the early years of the HIV epidemic traced the expansion of the field and changes of focus [36]. Additional studies captured the presence of new scientific terminology and the specialization of journals as the field progressed [37]–[39]. The emergence of the study of HIV as an interdisciplinary field of research, coupled with the advancement of scientometric analysis methods in recent years has enabled researchers to better assess collaboration patterns, geographic distribution, and expansion of subject areas [40], [41]. A recent evaluation of six NIH HIV/AIDS clinical trials networks showed that US-based authors collaborated with authors in 41 different countries on a total of 243 papers [42].

Different from previous studies that focused on simple statistics on the productivity of areas and individuals in terms of papers published, our study focused on patterns of collaboration using comprehensive network analysis methods. Additionally, we investigated the impact of international collaboration patterns on the population sample size of studies. From a global perspective, our study was also able to identify many countries for which no HIV and HPV estimates could be found. One of the limitations of our analysis is the scarce number of studies available. Different from other co-authorship network analyses using a more sensitive search strategy in Web of Science [4], [17], for two reasons we opted to have a more specific search strategy in Medline, Embase, Cochrane Library, and Global Health databases. Firstly, a more selective sample of studies made it feasible to manually extract data on the sample size of the studies and the origin of participants. Secondly, the databases used in our analysis are more specific for the medical literature.

The research networks presented in our paper are likely to be the intersection of both HIV and HPV research networks. Future studies should try to expand the analysis in order to jointly analyse HIV, HPV and co-infection research networks. As more data become available, it would also be beneficial to analyze their evolution over time. Statistics on research collaboration networks could be further correlated to information on research funding calls, public-private partnerships, global burden of disease and diplomatic agreements. Additionally, it would be interesting to evaluate the determinants of researchers' inclination to connect to different research groups. This analysis could be coupled with an evaluation of networks' structural holes [43]. Further investigations could also investigate the existence and the role of the Big-fish-small-pond effect [44] in epidemiological research networks.

International research networks not only can generate more precise epidemiological estimates for different countries, but they can also assist in knowledge transfer between developed and developing countries, as well as standardizing measurements and reducing duplication of research [45]–[47]. Moreover, network analysis can be used to monitor strategic goals such as integration and collaboration within and across research areas over time [19], [42]. Collaborative and coordinated efforts among those working in epidemiological studies worldwide are crucial in defining and implementing global health initiatives that will improve lives in both developed and developing countries.
